# Could titanium screws be an appropriate disc stabilization technique in temporomandibular joint arthroscopy?

**DOI:** 10.1007/s00784-024-05737-9

**Published:** 2024-06-18

**Authors:** Rafael Martin-Granizo, Luis Vicente González, Juan Pablo López

**Affiliations:** 1 Department of Oral and Maxillofacial Surgery, Hospital Clínico San Carlos, Madrid, Spain; 2 Oral and Maxillofacial Surgeon, Hospital Universitario La Samaritana, Bogotá, DC Colombia; 3 Department of Oral Research, School of Dentistry, Institución Universitaria de Colegios de Colombia UNICOC, Bogotá, DC Colombia; 4 Oral and Maxillofacial Surgery Surgeon, Hospital Universitario Fundación Santa Fe de Bogotá, Bogotá, DC Colombia; 5 Universidad El Bosque, Unidad de Investigación en Epidemiología Clínica Oral UNIECLO, Bogotá, DC Colombia

**Keywords:** Operative Arthroscopy, Temporomandibular joint, TMJ discopexy, Complications

## Abstract

Multiple techniques for disc fixation through temporomandibular joint arthroscopy have been described. They can be classified as non-rigid, semi-rigid, and rigid. They all offer different advantages and disadvantages, and some have greater difficulties than others. Currently, multiple modifications to the basic techniques have been described in order to facilitate the technique since disc fixation corresponds to one of the procedures that most require skill. However, each technique requires extensive evaluation and monitoring in order to avoid complications and find the benefits of each technique. For this reason, the objective of this letter to the editor is to discuss two situations observed in the previously described fixation technique with osteosynthesis screws. The first issue is the fixation mechanism, and the second is the fixation time. This is in order to continue searching for the truth among all to achieve the best results and the benefit of patients.

## Editor letter (Debate)

Dear Editor: We would like to congratulate the authors of the article “Arthroscopic reduction and rigid fixation of the previously displaced temporomandibular joint disc without reduction using a titanium screw: a case series.” [1] It is interesting to read articles with new ideas that seek to facilitate procedures that require a long learning curve. The minimally invasive surgical approach has become the trend for managing temporomandibular disc displacement. Many details of this surgical procedure were presented in this paper, and it is essential to set up a discussion.

The significance of disc repositioning and fixation in managing temporomandibular disc displacement is widely acknowledged today. However, the field offers many techniques with unique approaches and advantages. These techniques can generally be categorized into non-rigid, semi-rigid, and rigid fixation methods. Non-rigid techniques involve fixation to the capsule in the lateral portion, offering a degree of flexibility in disc positioning. On the other hand, semi-rigid methods entail fixation to the cartilage portion of the ear canal, which enhances the traction vector and aids in achieving optimal disc alignment. Finally, rigid techniques utilize titanium anchors or resorbable pins to provide robust stability during fixation, ensuring long-term efficacy. While disc fixation remains one of the more challenging procedures in our specialty, innovations and refinements to the basic techniques continue to emerge, simplifying the process and improving outcomes.

Among these techniques, rigid discopexy is an excellent option for patients. This approach utilizes titanium anchors with sutures to retract the disc into a more favorable position and offers notable advantages. Compared with open arthroplasty, Mitek anchors features represent a smooth surface and two winged arcs made of nickel-titanium alloy with shape memory superelasticity, ensuring reliable retention and stability within the medular condyle without any structural modification [2]. Indeed, titanium anchors have shown promise in achieving secure fixation of the TMJ disc following discopexy. However, it’s essential to recognize the nuanced differences between anchor-based methods and those employing titanium screws relative to the screw configuration (thread), as these distinctions may impact treatment outcomes and patient experiences, necessitating further exploration and consideration.

The biocompatibility, tissue response, and long-term implications must be addressed. Titanium screws have been extensively used in orthopedic and maxillofacial surgeries due to their excellent biomechanical properties and biocompatibility. However, concerns persist regarding potential mandibular condylar reactions in the short and long term. Osteosynthesis with titanium, despite its high tissue compatibility, has also been the cause of study due to the possibility of constant mechanical irritation that can cause serious condylar resorption and pseudoarthrosis [3]. Besides, considering that the screw has threads on their surfaces and could stimulate bone reabsorption as a response over time due to mechanic compression. The literature describes the mandibular head condyle reabsorption after condylar fracture reduction with a titanium screw of condylar fracture making emphasis on increased functional loading [4]. 

On the other hand, the post-surgical follow-up period is crucial to evaluate the outcome in these patients. We consider in this period essential factors that could influence the choice between and absorbable pins, titanium anchors or titanium screws based on literature studies. After approximately two years, the resorbable pins disappear from the condyles through gradual resorption after completing their stabilization. The titanium anchors do not have threads, and their retention is mechanical without increasing compression, both techniques have adecuatly follow-up publisehd. Finally, titanium screws do not have an adequate follow-up time at the moment. In contrast, the author presents the outcomes as a final result with just six months of follow-up period, which is too early to set a conclusion in this technique with a titanium screw. The follow-up period for the evaluation and approval of a surgical technique is of utmost importance since in arthroscopy, the changes that occur during evolution have already been demonstrated. The first few months may not be so promising, but they will be satisfying in the long-term. Furthermore, the consequences of a technique should be measured in the long-term [5]. 

In conclusion, the fixation technique shows efficacy in achieving stable disc fixation. However, several factors must be carefully weighed when selecting the appropriate technique for individual patients. Future research on long-term outcomes, biomechanical properties, and patient-reported outcomes will guide clinical decision-making and optimize treatment strategies for TMJ disorders.

## Data Availability

No datasets were generated or analysed during the current study.
